# Frequency-Domain Techniques for Cerebral and Functional Near-Infrared Spectroscopy

**DOI:** 10.3389/fnins.2020.00300

**Published:** 2020-04-07

**Authors:** Sergio Fantini, Angelo Sassaroli

**Affiliations:** Department of Biomedical Engineering, Tufts University, Medford, MA, United States

**Keywords:** near-infrared spectroscopy, diffuse optical imaging, frequency domain, cerebral hemodynamics, brain activation, fast optical signal, depth sensitivity

## Abstract

This article reviews the basic principles of frequency-domain near-infrared spectroscopy (FD-NIRS), which relies on intensity-modulated light sources and phase-sensitive optical detection, and its non-invasive applications to the brain. The simpler instrumentation and more straightforward data analysis of continuous-wave NIRS (CW-NIRS) accounts for the fact that almost all the current commercial instruments for cerebral NIRS have embraced the CW technique. However, FD-NIRS provides data with richer information content, which complements or exceeds the capabilities of CW-NIRS. One example is the ability of FD-NIRS to measure the absolute optical properties (absorption and reduced scattering coefficients) of tissue, and thus the absolute concentrations of oxyhemoglobin and deoxyhemoglobin in brain tissue. This article reviews the measured values of such optical properties and hemoglobin concentrations reported in the literature for animal models and for the human brain in newborns, infants, children, and adults. We also review the application of FD-NIRS to functional brain studies that focused on slower hemodynamic responses to brain activity (time scale of seconds) and faster optical signals that have been linked to neuronal activation (time scale of 100 ms). Another example of the power of FD-NIRS data is related to the different regions of sensitivity featured by intensity and phase data. We report recent developments that take advantage of this feature to maximize the sensitivity of non-invasive optical signals to brain tissue relative to more superficial extracerebral tissue (scalp, skull, etc.). We contend that this latter capability is a highly appealing quality of FD-NIRS, which complements absolute optical measurements and may result in significant advances in the field of non-invasive optical sensing of the brain.

## Introduction

Diffuse optical imaging, in the near-infrared spectral band (wavelength range: 600–1000 nm), affords non-invasive sensing of the human brain through the intact scalp and skull. The associated instrumentation can be sufficiently lightweight and compact to be applicable at the bedside, in an ambulance, on the field (accident scene, sports venue, battlefield, etc.), in every-day settings, and even worn by subjects while they engage in normal activities. These enormous practical advantages of non-invasive diffuse optical imaging, combined with a strong sensitivity to cerebral hemodynamics and oxygenation, compensate intrinsic limitations in penetration depth and spatial resolution, and account for the large number of applications and the growing interest in this technology. Several review articles cover a variety of topics in cerebral near-infrared spectroscopy (NIRS), including a historical overview (Ferrari and Quaresima, 2012), description of instrumentation and methodology (Scholkmann et al., 2014b), clinical applications (Irani et al., 2007; Smith, 2011; Obrig, 2014; Yang et al., 2019), brain oximetry in newborns and adults (Wolf et al., 2012; Nielsen, 2014; la Cour et al., 2018), and critical perspectives (Fantini et al., 2018; Quaresima and Ferrari, 2019).

NIRS can be performed using methods in continuous wave (CW) (constant illumination), time domain (TD) (pulsed illumination and time-resolved detection), or frequency domain (FD) (intensity-modulated illumination and phase-resolved detection). Review articles have been devoted to functional NIRS (fNIRS) based on CW methods (Scholkmann et al., 2014b), the most common technique and the one embraced by almost all commercial instruments, and TD methods (Torricelli et al., 2014). No review articles have been previously devoted specifically to cerebral and functional FD-NIRS, and this article intends to fill this gap. However, the general topic of FD-NIRS and its applications to biological tissue are covered in book chapters (Fantini et al., 1997; Cerussi and Tromberg, 2003; Srinivasan et al., 2011; Fantini and Sassaroli, 2016), review and tutorial articles (Chance et al., 1998b; O’Sullivan et al., 2012), and extended sections in biomedical optics books (Tuchin, 2007; Wang and Wu, 2007; Bigio and Fantini, 2016, Chapter 12).

As mentioned, CW techniques are by far the most commonly used, especially in the commercial space; in fact, to the best of our knowledge, while more than ten companies manufacture CW-NIRS systems worldwide, at the time of writing only one company (Hamamatsu Photonics, Japan) manufactures TD-NIRS systems, and only one company (ISS, Inc., United States) manufactures FD-NIRS systems. In the research arena, however, TD-NIRS and FD-NIRS are employed by several groups worldwide.

One question that is investigated in this article is the following: what is the advantage of using FD techniques for cerebral and functional NIRS, given its added instrumental complexity compared to CW-NIRS? This question will be explored by first describing the information content of FD data and how it enriches and differs from that of CW data, and then by describing the way in which FD data have been used in the field of NIRS studies of the brain. Following this description and a review of the literature, we will express our views on the potential role of FD techniques in cerebral and functional NIRS as this field is growing and evolving.

## Frequency-Domain Near-Infrared Spectroscopy (FD-NIRS)

### The FD-NIRS Data: DC Intensity, AC Amplitude, Phase

The basic idea of FD-NIRS is to use intensity-modulated illumination and phase-sensitive detection. Sinusoidal modulation, or a sinusoidal component of the modulated signal, at a given angular frequency ω is typical considered, so that the time-dependence of the FD-NIRS signal is described by *e*^−*iωt*^. As a result, the optical signal measured in FD-NIRS consists of an average, or direct current (DC), intensity, and by a phasor described by an amplitude, or alternating current (AC) intensity, and a phase (*φ*):

(1) $$FD\text{-}NIRSsignal=DC+ACe^{i\varphi }e^{-i\omega t}$$

Typical modulation frequencies employed in the application of FD-NIRS to biological tissue are of the order of 100 MHz. Such a choice is related to the photon mean time of flight (*t*), which is of the order of nanoseconds for light propagation in typical biological tissues and for distances of centimeters between the illumination and detection points. Since the phase is related to the mean time of flight by the relationship *φ* ≈ *ωt*, a measurable phase of the order of 1 rad requires a modulation frequency [*f* = *ω*/(2*π*)] of the order of 100 MHz. Lower modulation frequencies (say, 10 MHz or less) would cause a smaller phase shift that may be close to the limit of phase detectability. On the contrary, higher modulation frequencies (say, 1 GHz or more) would result in smaller AC amplitudes, possibly below the noise level, because of an increasing attenuation of the FD phasor at higher frequencies.

The solution of the diffusion equation in the frequency domain shows that the energy density distribution in turbid media resulting from intensity-modulated illumination can be described in terms of photon-density waves (PDWs) (Fishkin and Gratton, 1993; Tromberg et al., 1993). For a point-source in an infinite medium, PDWs are overdamped spherical waves. For typical optical properties of biological tissue in the near-infrared (absorption coefficient: *μ*_*a*_ = 0.1 *cm*^−1^; reduced scattering coefficient: $\mu_{s}^{\prime }=10cm^{-1}$), and for a typical modulation frequency of 100 MHz, the wavelength of the photon density waves is about 25 cm, while its attenuation length (i.e., the distance over which the energy density decreases by a factor of *e*) is about 0.6 cm. Therefore, PDWs are observable only over a fraction of their wavelength, rendering them analogous to near field waves (Bigio and Fantini, 2016, Section 12.4).

For typical modulation frequencies used in FD-NIRS and for typical optical properties of tissue in the near-infrared, DC intensity and AC amplitude carry a similar information content. For example, they feature a similar decay at increasing distances from the light source (although the AC attenuation is stronger than the DC attenuation), and a similar dependence to localized or homogeneous changes in the optical properties of tissue. However, the AC amplitude offers the practical advantage of being relatively insensitive to room light, or to any non-modulated stray light, so that it is often used instead of DC intensity when data are collected with FD-NIRS instruments.

### Instrumentation for FD-NIRS

A review of basic instrumentation aspects of FD-NIRS can be found in Chance et al. (1998b) and in Bigio and Fantini, (2016), Section 13.4.2). A description of FD-NIRS instruments used in a variety of applications is beyond the scope of this review article, which specifically focuses on non-invasive brain measurements. The interested reader is referred to the description of FD-NIRS instruments based on homodyne detection (Yang et al., 1997; Ramanujam et al., 1998; Cheung et al., 2001; Yu et al., 2003), heterodyne detection (Fantini et al., 1995; Ramanujam et al., 1998; McBride et al., 2001; Nissilä et al., 2002, 2005), and on a radiofrequency network analyzer (Pham et al., 2000; Gulsen et al., 2006).

Recent developments in FD-NIRS instrumentation include implementations based on a compact frequency-sweeping circuit board (No et al., 2008), digital heterodyning (Arnesano et al., 2012), direct digital sampling (Roblyer et al., 2013; Zimmermann et al., 2016), frequency division multiplexing (Torjesen et al., 2017; Zhao et al., 2018), CMOS integrated circuitry (Sthalekar and Joyner Koomson, 2013; Yun and Joyner Koomson, 2013), and vertical cavity surface emitting lasers (VCSEL) (Sultan et al., 2013; Kitsmiller et al., 2018).

With regards to commercially available FD-NIRS instruments, they have only been made available by NIM, Inc. (Philadelphia, PA, United States), which operated from 1996 to 2009 (Ferrari and Quaresima, 2012), and by ISS, Inc. (Champaign, IL, United States), which introduced its first FD-NIRS instrument in the late 1990’s and now offers FD-NIRS instruments for quantitative spectroscopy, oximetry, and imaging. Most non-invasive FD-NIRS studies of the brain are currently performed with ISS instruments, which operate by parallel multi-channel heterodyne detection and temporal multiplexing of light sources.

### Measurement of Absolute Optical Properties With FD-NIRS

Diffusion theory yields analytical expressions of the FD-NIRS signals collected in a semi-infinite geometry. In this case, the light source and the optical detector are located on the tissue boundary, and the tissue is considered to be a uniform, semi-infinite medium, with optical properties described by an absorption coefficient (*μ*_*a*_) and a reduced scattering coefficient ($\mu_{s}^{\prime }$). Such analytical solutions show that one can define functions of measured DC, AC, and phase signals (*F*_*DC*_, *F*_*AC*_, *F*_*φ*_, respectively) that are linearly dependent on the distance (*r*) between the source and the detector [as one may derive, for example, from Eqs. (12.33), (12.35), and (12.36) in Bigio and Fantini (2016; Section 12.6)], even though actual measurements of such functions of DC, AC, and phase are also affected by instrumental terms associated with the source and the detector:

(2) $$\begin{array}{c} F_{DC}\left(DC,\rho ,\mu_{a},\mu_{s}^{{}^{\prime }}\right)\\ \;=S_{DC}\left(\mu_{a},\mu_{s}^{{}^{\prime }}\right)\rho +K_{DC-Source}+K_{DC-Detector} \end{array}$$

(3) $$\begin{array}{c} F_{AC}\left(AC,\rho ,\omega ,\mu_{a},\mu_{s}^{{}^{\prime }}\right)\\ \;=S_{AC}\left(\omega ,\mu_{a},\mu_{s}^{{}^{\prime }}\right)\rho +K_{AC-Source}+K_{AC-Detector} \end{array}$$

(4) $$\begin{array}{c} F_{\varphi }\left(\varphi ,\rho ,\omega ,\mu_{a},\mu_{s}^{{}^{\prime }}\right)\\ \;=S_{\varphi }\left(\omega ,\mu_{a},\mu_{s}^{{}^{\prime }}\right)\rho +K_{\varphi -Source}+K_{\varphi -Detector} \end{array}$$

In Eqs. (2–4), *S*_*DC*_, *S*_*AC*_, *S*_*φ*_ are the slopes of the linear functions of *r*, and the additive *K* terms indicate instrumental factors contributed by the source or the detector to the DC, AC, or phase measurement, as indicated by the subscripts. While the *K* terms are unknown, as they are determined by several instrumental factors as well as by the optical coupling of the source and the detector with tissue, the functions *F* and the slopes *S* are known from diffusion theory. The functions *F* can be approximated with $F_{DC}\left(DC,\rho ,\mu_{a},\mu_{s}^{\prime }\right)=ln\left(\rho^{2}DC\right)$, $F_{AC}\left(AC,\rho ,\mu_{a},\mu_{s}^{\prime }\right)=ln\left(\rho^{2}AC\right)$, and $F_{\varphi }\left(\varphi ,\rho ,\omega ,\mu_{a},\mu_{s}^{\prime }\right)=\varphi$ if the condition $\rho \sqrt{3\mu_{a}\mu_{s}^{\prime }}\gg 1$ is fulfilled (Fantini et al., 1999). Equations (2–4) provide a basic description of how FD-NIRS can accomplish absolute measurements of optical properties according to different methods that are described below.

#### Multi-Distance Methods

Multi-distance methods rely on measurements at multiple (at least two) source-detector distances (*ρ*), typically within a range of 1.5–4 cm. The goal is to measure the slopes [*S*_*DC*_, *S*_*AC*_, *S*_*φ*_ in Eqs. (2–4)], which only depend on the optical properties (and the known modulation frequency) through analytical expressions (at least for homogeneous media) that can be inverted to yield absolute values of *μ*_*a*_ and $\mu_{s}^{\prime }$. Two data types (either DC and *φ*, or AC and *φ*) are needed to determine the two optical coefficients (Fantini et al., 1994a). The solution of the diffusion equation for semi-infinite media with extrapolated boundary conditions (Haskell et al., 1994) has been used in an iterative approach that accounts for the presence of *μ*_*a*_ and $\mu_{s}^{\prime }$ on the left-hand side of Eqs. (2–4) (Fantini et al., 1994b). As mentioned above, under the assumption $\rho \sqrt{3\mu_{a}\mu_{s}^{\prime }}\gg 1$ one may use approximate expressions for *F*_*DC*_, *F*_*AC*_, and *F*_*φ*_ (namely ln (*ρ*^2^*DC*), ln (*ρ*^2^*AC*), and *φ*) that offer the advantage that *μ*_*a*_ and $\mu_{s}^{\prime }$ do not appear on the left-hand side of Eqs. (2–4) (Fantini et al., 1999). We observe that in the case of non-invasive NIRS measurements of the brain (for which *μ*_*a*_ and $\mu_{s}^{\prime }$ are of the order of 0.1 cm^–1^ and 8 cm^–1^, respectively) the condition $\rho \sqrt{3\mu_{a}\mu_{s}^{\prime }}\gg 1$ starts becoming fulfilled at source-detector distances *ρ* of about 2 cm, for which $\rho \sqrt{3\mu_{a}\mu_{s}^{\prime }}\approx 2$, as we verified by comparing the results based on this approximation with those obtained with an iterative approach applied to the full solution of the diffusion equation.

Equations (2–4) clarify the importance of the source-detector configuration used in multi-distance methods. If the configuration uses a single detector and multiple sources placed at different distances from it, then the *K*_*Detector*_ terms in Eqs. (2–4) are all independent of *ρ* (i.e., they are the same for all source-detector pairs) and therefore they cancel out in the determination of the slopes *S*. By contrast, the *K*_*Source*_ terms associated with each of the multiple sources are all different and must somehow be determined by a preliminary calibration on a tissue-like phantom with known optical properties. A reciprocal argument applies to the case of a configuration that uses a single source and multiple detectors (in which case, of course, it is the *K*_*Detector*_ terms that are different for each source-detector pair). In a case where one uses a single source and a single detector, so that multi-distance measurements are accomplished by scanning the source and/or detector relative to each other, no preliminary calibration is needed, since both *K*_*Source*_ and *K*_*Detector*_ terms are the same for all measurements at different values of ρ (under the assumption that the optical coupling with tissue is kept constant throughout the scanning of source or detector). While this latter approach is the most robust for measurements in a lab setting, especially on tissue-like phantoms (Fantini et al., 1994b; Hallacoglu et al., 2013; Applegate and Roblyer, 2018), it is not practical for *in vivo* measurements, for which either multiple sources or multiple detectors are typically used. It is very important to observe that the multi-distance methods described above require that the optical coupling be the same in the calibration phantom and in tissue (for the method based on multiple sources or multiple detectors), or at the multiple source-detector distances considered (for the methods based on scanning the single source or single detector). This is an issue of high practical importance, and a potential limitation that can be addressed by the multi-distance method described next.

An ingenious, yet simple multi-distance method for absolute measurements of optical properties that does not require any preliminary calibration and does not require assumptions about optical coupling with tissue is the self-calibrating method (Hueber et al., 1999). This method uses two sources and two detectors that are arranged in such a way to generate data described by two sets of Eqs. (2–4) where the *K*_*Source*_ and *K*_*Detector*_ terms have opposite signs, and thus can be canceled out by taking the average of the two sets of equations (Hueber et al., 1999). This method corrects for both instrumental and optical coupling contributions to the *K*_*Source*_ and *K*_*Detector*_ terms, thus minimizing potential confounding effects related to instrumental drifts, variable optical coupling with tissue, and motion artifacts.

#### Single-Distance Methods

One possible approach to absolute measurements with single-distance FD-NIRS data is to perform a preliminary calibration on a phantom with known optical properties to determine the term *K*_*Source*_ + *K*_*Detector*_ in Eqs. (2–4). While in principle this method is straightforward, it relies on a much more limiting assumption than the calibration methods for multi-distance measurements. While multi-distance calibrations assume that the *relative* optical coupling at the various distances is the same on the calibration phantom and on tissue, the single-distance calibration assumes that the *absolute* optical coupling is the same on phantom and tissue. This is a much stronger assumption that is difficult to realize in practice.

A more robust method for absolute single-distance measurements in the frequency domain is a multi-frequency approach. This method is also based on the solution of the diffusion equation represented by Eqs. (2–4). However, while in multi-distance methods the independent variable is the source-detector distance, *ρ* (i.e., FD-NIRS data are measured at several source-detector distances), in the multi-frequency approach the independent variable is the modulation frequency, *ω* (i.e., FD-NIRS data are measured at several modulation frequencies). Usually the modulation frequency is swept from tens to hundreds of MHz, while the source-detector distance is fixed at about 3 cm (Pham et al., 2000; Gulsen et al., 2006). Of course, any dependence on *ω* of the *K*_*Source*_ and *K*_*Detector*_ terms must be considered through calibration measurements on a reference phantom or at multiple source-detector distances (Bevilacqua et al., 2000; Pham et al., 2000). Iterative methods based on non-linear least-squares fits have been used for the recovery of the optical properties in the multi-frequency approach (Haskell et al., 1994).

### Measurement of Absorption Changes With FD-NIRS

Most non-invasive optical studies of the brain aim to detect absorption changes related to cerebral hemodynamic and oxygenation changes. One possible exception is the measurement of the fast optical signal, which will be presented in Section 3.6. In the case of a homogeneous absorption change, *Δμ*_*a*_, in a semi-infinite medium, one can translate relative changes in the DC Intensity (*ΔI*_*DC*_/*I*_*DC*0_, where we indicate with *I*_*DC*0_ the detected DC intensity in a reference condition, say at baseline, and with *ΔI*_*DC*_ the intensity change with respect to the reference condition as a result of an absorption change *Δμ*_*a*_) and absolute changes in the phase (*Δφ*) into the corresponding absorption change as follows (Blaney et al., 2020; Fantini et al., 2020):

(5) $$\Delta \mu_{a}\left(I_{DC}\right)=-\frac{2\left(1+\rho \sqrt{3\mu_{a0}\mu_{s0}^{\prime }}\right)}{3\rho^{2}\mu_{s0}^{\prime }}\left(\frac{\Delta I_{DC}}{I_{DC0}}\right)$$

(6) $$\Delta \mu_{a}\left(\varphi \right)=-\left(\frac{1+\frac{\sqrt{\gamma +1}}{\gamma \rho }\sqrt{\frac{2}{3\mu_{a0}\mu_{s0}^{\prime }}}+\frac{1}{3\mu_{a0}\mu_{s0}^{\prime }\gamma \rho^{2}}}{\rho \frac{\sqrt{\gamma -1}}{2\gamma }\sqrt{\frac{3\mu_{s0}^{\prime }}{2\mu_{a0}}}}\right)\Delta \varphi$$

where the subscripts 0 indicate a reference condition for which *μ*_*a*_ = *μ*_*a*0_, the changes with respect to this reference condition are *Δμ*_*a*_ = *μ*_*a*_ − *μ*_*a*0_, *ΔI*_*DC*_ = *I*_*DC*_ − *I*_*DC*0_, *Δφ* = *φ* − *φ*_0_, and $\gamma =\sqrt{1+{\left(\frac{\omega }{c_{n}\mu_{a0}}\right)}^{2}}$, with *c*_*n*_ speed of light in tissue. Equation (5) represents the modified Beer-Lambert law that is extensively used in CW-NIRS (Delpy et al., 1988; Sassaroli and Fantini, 2004). A correction to Eq. (5) is needed for the AC amplitude of intensity (*I*_*AC*_); such correction can be found in Sassaroli et al. (2019) and Blaney et al. (2020). The effect of localized absorption changes on the DC Intensity (and similarly on the AC amplitude) and on the phase can be described in terms of spatial regions of sensitivity, which are introduced in Section “The Spatial Region of Sensitivity of DC, AC, and Phase.”

In the case of high modulation frequencies, such that *ω*≫*c*_*n*_*μ*_*a*_, one can show that the term in parenthesis on the right-hand side of Eq. (6) becomes independent of *μ*_*a*0_ so that, under the further assumption that scattering is independent on wavelength, the ratio of phase changes at two wavelengths *λ*_*1*_ and *λ*_*2*_ equals the ratio of absorption changes at the same wavelengths:

(7) $$\frac{\Delta \varphi \left(\lambda_{1}\right)}{\Delta \varphi \left(\lambda_{2}\right)}=\frac{\Delta \mu_{a}\left(\varphi ,\lambda_{1}\right)}{\Delta \mu_{a}\left(\varphi ,\lambda_{2}\right)}$$

In the ideal case of a baseline condition that features no absorption (i.e., *μ*_*a*0_ = 0), *Δμ*_*a*_ = *μ*_*a*_ and one can write (Sevick et al., 1991):

(8) $$\frac{\Delta \varphi \left(\lambda_{1}\right)}{\Delta \varphi \left(\lambda_{2}\right)}=\frac{\mu_{a}\left(\varphi ,\lambda_{1}\right)}{\mu_{a}\left(\varphi ,\lambda_{2}\right)}$$

This result may be used to quantify the oxygen saturation of hemoglobin associated with an absorption change from baseline, but the condition *ω*≫*c*_*n*_*μ*_*a*_ and the assumption $\mu_{s0}^{\prime }\left(\lambda_{1}\right)≅\mu_{s0}^{\prime }\left(\lambda_{2}\right)$ are somewhat limiting. In fact, for *μ*_*a*_ = 0.05 cm^–1^, one would need to use a modulation frequency [*f* = *ω*/(2*π*)] that is much greater than ∼170 MHz.

### The Spatial Region of Sensitivity of DC, AC, and Phase

In this section, we describe the spatial region in tissue that is probed in FD-NIRS by a given source-detector pair that is placed on the tissue surface. This description requires several specifications. In fact, (1) different data types (DC, AC, *φ*) have different regions of sensitivity; (2) the sensitivity itself is not constant within the overall probed volume, so that a given optical perturbation in some portions of the probed region induce stronger changes in the optical signals than others; (3) the region of sensitivity can be different for different kinds of optical contrast, say for an absorption vs. a scattering perturbation. Here, we consider localized changes within a small region (conceptually a point-like region), in either the absorption coefficient or reduced scattering coefficient, in an otherwise optically homogeneous medium. The case of an absorption perturbation has been studied in two opposite regimes: in the limit of infinitely absorbing defects (Feng et al., 1995) and in the limit of infinitesimal changes in the absorption coefficient (Born approximation) (Boas, 1997). The relationship between these two extreme approaches has been recently investigated (Sassaroli et al., 2014). For the case of a scattering perturbation, only the Born approximation has been considered (Martelli et al., 2010). The region of sensitivity for a specific optical measurement with respect to a local change in optical properties also depends on the background optical properties, the shape of tissue and the nature of boundary conditions, and the spatial arrangements of source(s) and detector(s). If we denote with *Y* any optical measurement (for example, DC, AC, or phase in FD-NIRS), we define a dimensionless sensitivity of such a measurement to a point-like perturbation in a given optical coefficient *μ* (which may be either *μ*_*a*_ or $\mu_{s}^{\prime }$) as follows:

(9) $$S_{Y,\mu }\left(r\right)=\frac{\left(\frac{\partial Y}{\partial \mu_{r}}\right)}{\left(\frac{\partial Y}{\partial \mu }\right)}$$

where the derivative in the numerator is taken with respect to a point-like perturbation at position **r** (*μ*_**r**_), whereas the derivative in the denominator is taken with respect to a uniform change, everywhere in the medium, in the optical property *μ*.

**FIGURE 1:**
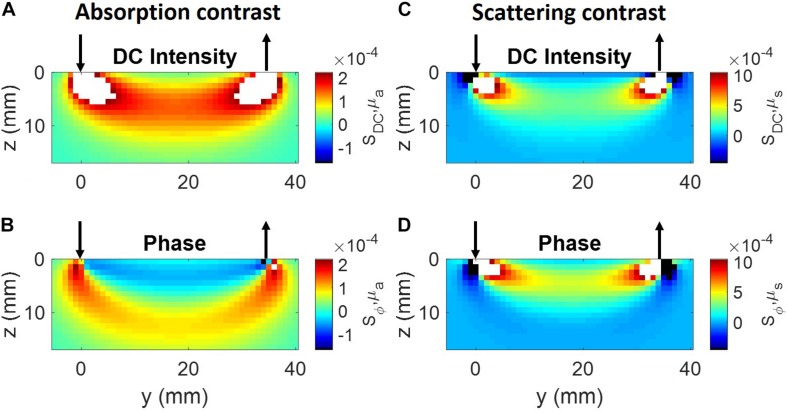
Sensitivity maps in the (*y.*z) plane, where the tissue boundary is the *x-y* plane, the source (arrow pointing down) is at (0,0,0), and the detector (arrow pointing up) is at (0, 35 mm, 0). **(A)** DC Intensity, absorption contrast; **(B)** phase, absorption contrast; **(C)** DC intensity, scattering contrast, **(D)** phase, scattering contrast. The color bar labels in panels **(A,C)** indicate the sensitivity of DC intensity with respect to absorption (*S*_*DC*,*μ*_*a*__) and reduced scattering coefficients (*S*_*DC*,*μ*_*s*__), respectively. The color bar labels in panels **(B,D)** indicate the sensitivity of phase with respect to absorption (*S*_*ϕ*,*μ*_*a*__) and reduced scattering coefficients (*S*_*ϕ*,*μ*_*s*__), respectively. White and black in the color maps indicate values greater than the maximum or smaller than the minimum, respectively, of the color bars.

We have calculated the spatial sensitivity defined in Eq. (9) using diffusion theory for a semi-infinite medium of optical properties *μ*_*a*_ = 0.1 *cm*^−1^ and $\mu_{s}^{\prime }=10cm^{-1}$, a single source-detector pair placed on the medium boundary at a source-detector distance of 35 mm, and an optical perturbation 1 mm^3^ in size. The modulation frequency was 140 MHz. The results are reported in Figure 1 for DC intensity (top panels) and phase (bottom panels), and for perturbations in absorption (left panels) or scattering (right panels). Positive sensitivity values at any position **r** mean that the considered data changes in the same direction (increase or decrease) as a result of a localized change at **r** or a homogeneous change in the considered optical property. Figure 1 shows that the DC intensity sensitivity (i.e., the sensitivity in CW-NIRS) is always positive, for both absorption and scattering perturbations (with a possible exception of scattering defects near the source or the detector), and has a typical banana shape (Feng et al., 1995). Also, DC intensity sensitivity is especially high for optical perturbations close to the source and the detector. The phase sensitivity is more complex. In the case of absorption perturbations, it features a “double banana,” a shallower one of negative sensitivity and a deeper one of positive sensitivity. In the case of scattering perturbations, the phase sensitivity is mostly positive, and also banana shaped, except for small regions close to the source and the detector. Based on Figure 1, one can appreciate the different nature of the optical sensitivity of intensity and phase data to absorption and scattering contrast.

## Use of FD-NIRS for Non-Invasive Brain Studies

### Imaging With Phased Arrays

In Section “The FD-NIRS Data: DC Intensity, AC Amplitude, Phase,” we mentioned that the photon-density waves generated by the intensity-modulated light sources in FD-NIRS are near-field waves. They are only measurable within a fraction of a wavelength. This is in contrast with the case of ultrasound imaging, where the ultrasound wavelength in soft tissue is typically of the order of hundreds of microns, and phased arrays are commonly used to focus and steer ultrasound beams. Nevertheless, phased arrays have also been proposed in FD-NIRS to exploit the interference of photon-density waves (Schmitt et al., 1992). In particular, it was proposed to achieve the accurate localization of small defects by scanning the null-plane generated by two light sources that are modulated in opposition of phase (Chance et al., 1993; Chance et al., 1996). This method has been applied to the study of sensorimotor (finger touching) and cognitive (backward spelling) activation in human subjects, demonstrating a high signal-to-noise ratio (>40) of phase measurements, resulting from the large (180 degrees) phase transition at the null plane (Chance et al., 1998a). The phased-array method has been used as a basis for diffuse optical tomography (Intes et al., 2002; Rajan et al., 2008), and it was proposed to implement it by post-processing CW-NIRS data (Liu et al., 2005a,b). The emphasis of the phased array approach is on an improvement of the spatial localization and resolution in diffuse optics. Because such an improvement comes at the expense of a more complex instrumentation, targets specific classes of dynamic perturbations, and introduces some limitations to the field of view, it has not found broad applicability in diffuse optics. Of course, this does not mean that it may not be implemented in such a way to benefit specific applications in the near future.

### Absolute Measurements With a Combination of Intensity and Phase Data

A common use of time-resolved NIRS, in either the time domain or the frequency domain, is toward measurements of absolute optical properties of tissue, namely its absorption (*μ*_*a*_) and reduced scattering ($\mu_{s}^{\prime }$) coefficients. Measurements at a minimum of two wavelengths, in conjunction with the Beer’s law relationship between absorption and chromophores concentration, allow for the translation of absorption coefficients into cerebral concentrations of oxy-hemoglobin ([HbO_2_]), deoxy-hemoglobin ([Hb]), total hemoglobin ([HbT] = [HbO_2_] + [Hb]), and hemoglobin saturation (StO_2_ = [HbO_2_]/[HbT]). Here, the “t” in StO_2_ indicates that the oxygen saturation of hemoglobin refers to an average over the investigated tissue. It is important to note that absolute measurements of StO_2_ may still be obtained from absorption coefficients that are only known to within an unknown factor, because StO_2_ depends on the ratio of absorption coefficients at multiple wavelengths (Bigio and Fantini, 2016, Section 15.1.3.1). This fact accounts for more robust absolute measurements of StO_2_ with respect to [Hb] or [HbO_2_], which instead rely on accurate absolute measurements of *μ*_*a*_. For this reason, it is possible that FD and CW measurements of StO_2_ (where the latter typically need to introduce some *ad hoc* assumptions on the scattering properties of tissue) may yield comparable results, as recently reported in a study on healthy human subjects during controlled hypoxia (Davies et al., 2017).

Multi-distance methods (see Section “Multi-Distance Methods”) have been employed for absolute measurements of cerebral concentration and/or saturation of hemoglobin in newborn piglets (Fantini et al., 1999; Zhang et al., 2000), in rats (Culver et al., 2003), in newborn and infants (Franceschini et al., 2007; Grant et al., 2009; Roche-Labarbe et al., 2010, 2012; Lin et al., 2013b, Lin T.-Y. et al., 2016; Dehaes et al., 2014, 2015; Demel et al., 2014b, 2015; Farzam et al., 2017; Ferradal et al., 2017; Schwarz et al., 2018), and in adults under normal conditions (Fantini et al., 2003; Gatto et al., 2006, 2007; McIntosh et al., 2010; Hallacoglu et al., 2012, 2013; Scholkmann et al., 2013a; Clancy et al., 2015; Kainerstorfer et al., 2015; Yang and Dunn, 2015; Moreau et al., 2016; Davies et al., 2017; Blaney et al., 2019; Pham et al., 2019), under anesthesia (Paisansathan et al., 2007; Meng et al., 2012a,b), hypoxia (Davies et al., 2017), pathological conditions such as multiple sclerosis (Yang and Dunn, 2015), stroke (Moreau et al., 2016), traumatic brain injury (Davies et al., 2019), before and during electro-convulsive therapy (ECT) (Fabbri et al., 2003), during neurovascular surgery (Calderon-Arnulphi et al., 2007), and after death (Gatto et al., 2006). The absolute values of [Hb], [HbO_2_], [HbT], and StO_2_ reported in these studies are summarized in Table 1 for piglets and rats, Table 2 for neonates and infants, and Table 3 for human adults. The absolute values of hemoglobin concentration and saturation reported in Tables 1–3 represent typical reported values (thus no error is provided) under baseline conditions at rest (unless otherwise noted).

**TABLE 1:** Absolute cerebral concentrations and oxygen saturation of hemoglobin in anesthetized newborn piglets and rats measured with non-invasive FD-NIRS *in vivo*.

References	Instr.	*f* (MHz)	Method	# of λ	[Hb] (μM)	[HbO_2_] (μM)	[HbT] (μM)	StO_2_ (%)	Age (days)	Animal model
Ntziachristos et al., 1997	NIM	200	SD (phase): ∼2 cm	2	–	–	–	80	1–5	Piglet
Fantini et al., 1999	ISS	110	MD: 1.5–3 cm	2	17	26	43	60	11 ± 1	Piglet
Zhang et al., 2000	ISS	110	MD: 1.5–3 cm	2	15	30	45	67	9 ± 2	Piglet
Culver et al., 2003	Own	70	MD: 0.3–1 cm	3	30	70	100	70	Adult	Rat

*Instr., instrument; NIM, NIM (Near Infrared Monitoring), Inc. (Philadelphia, PA, United States); ISS, ISS, Inc. (Champaign, IL, United States); SD, single distance; MD, multi distance.*

**TABLE 2:** Absolute cerebral concentrations and oxygen saturation of hemoglobin in neonates, infants, and children measured with non-invasive FD-NIRS *in vivo*.

References	Instr.	*f* (MHz)	Method	# of λ	[Hb] (μM)	[HbO_2_] (μM)	[HbT] (μM)	StO_2_ (%)	Age
Watzman et al., 2000	NIM	200	SD: 3 or 4 cm	3	–	–	–	73	0–6 years
Zhao et al., 2005	Own	140	SD: 4 cm	2	16	24	40	59	1–17 days
Franceschini et al., 2007	ISS	110	MD: 1–2.5 cm	7	17	35	52	67	1 week
					14	19	33	57	6 weeks
					21	32	53	61	12 weeks
					24	49	73	67	52 weeks
Grant et al., 2009	ISS	110	MD: 1–2.5 cm	7	21	39	60	65	0–15 days
Roche-Labarbe et al., 2010	ISS	110	MD: 1–2.5 cm	6	12	28	40	70	1 week
					12	18	30	60	6 weeks
Roche-Labarbe et al., 2012	ISS	110	MD: 1–2.5 cm	6	15	40	55	73	1 week
					15	28	43	65	10 weeks
Lin et al., 2013b	ISS	110	MD: 1–2.5 or 1.5–3 cm	6	19	41	60	69	3.6 ± 1.7 weeks
Dehaes et al., 2014	ISS	110	MD: 1–2.5 cm	6	17	45	62	72	0–4 days
Demel et al., 2014b	ISS	110	MD: 1.5–3 cm	2	15	23	38	60	2 days
Dehaes et al., 2015	ISS	110	MD: 1.5–3 cm	6	16	35	51	69	33–71 h
Demel et al., 2015	ISS	110	MD: 1.5–3 cm	2	15	24	39	62	7–71 h (preterm)
					18	30	48	63	7–54 h (term)
Lin P. Y. et al., 2016	ISS	110	MD: 1–2.5 cm	6	–	–	–	50–70	3–14 weeks (preterm)
Farzam et al., 2017	ISS	110	MD: 1.5–3 cm	8	19	41	60	69	2.4 ± 0.8 days (males)
					16	33	50	65	2.6 ± 1.5 days (females)
Ferradal et al., 2017	ISS	110	MD: 1.5–3 cm	8	26	40	66	60	∼4 days (anesthesia)
Schwarz et al., 2018	ISS	110	MD: 1.5-3 cm	2	–	–	–	54	4–13 days

*Instr., Instrument; NIM, NIM (Near Infrared Monitoring), Inc. (Philadelphia, PA, United States); ISS, ISS, Inc. (Champaign, IL, United States); SD, single distance; MD, multi distance.*

**TABLE 3:** Absolute cerebral concentrations and oxygen saturation of hemoglobin in human adults measured with non-invasive FD-NIRS *in vivo*.

References	Instr.	*f* (MHz)	Method	# of λ	[Hb] (μM)	[HbO_2_] (μM)	[HbT] (μM)	StO_2_ (%)	Age (y)	Notes
Fabbri et al., 2003	ISS	110	MD: 1.5–3 or 1–2.5 cm	2	14	19	33	57	27–57	Before ECT
Fantini et al., 2003	ISS	110	MD:	2	14	20	34	59	29–44	Before sleep
Gatto et al., 2006	ISS	110	MD: 2–4 cm	2	13	24	37	64	36 ± 9	
Gatto et al., 2007	ISS	110	MD: 2–4 cm	2	15	28	43	65	20–39	
					13	22	35	63	40–60	
Calderon-Arnulphi et al., 2007	ISS	110	MD: 2–3.5 cm	2	16	16	32	50	58–81	Before surgery
Paisansathan et al., 2007	ISS	110	MD: 2–4 cm	2	13	19	32	59	44 ± 14	Anesth.
McIntosh et al., 2010	ISS	110	MD: 2–3.5 cm	2	19	26	45	58	20–53	
Meng et al., 2012a	ISS	110	MD: 2–3.5 cm	2	11	30	41	73	22–68	Anesth.
Meng et al., 2012b	ISS	110	MD: 2–3.5 cm	2	11	25	36	68	22–68	Anesth.
Hallacoglu et al., 2012	ISS	110	MD: 2.8–3.8 cm	2	20	39	59	66	28 ± 4	
					18	22	40	55	85 ± 6	
Hallacoglu et al., 2013	ISS	110	MD: 1.3–4.8 cm	2	11	15	26	42	29 ± 2	Superf. tissue
					31	51	82	64		Deep tissue
					17	30	47	64		Homog. tissue
Scholkmann et al., 2013a	ISS	110	MD: 2–4 cm	2	–	–	–	59	–	
Clancy et al., 2015	ISS	110	MD	2	11	29	40	72	–	
Yang and Dunn, 2015	ISS	110	MD: 2–3.5 cm	2	17	28	45	63	50 ± 8	
Kainerstorfer et al., 2015	ISS	110	MD: 2–3.5 cm	2	16	35	51	69	21–50	
Moreau et al., 2016	ISS	110	MD: 2–3.5 cm	2	20	35	56	63	42 ± 7	
Davies et al., 2017	ISS	110	MD	2	–	–	–	62	–	
Davies et al., 2019	ISS	110	MD: 3–4.5 cm	2				67	50 ± 17	TBI
Blaney et al., 2019	ISS	140	MD: 1.1–4 cm	2	18	24	42	56	25–53	
Pham et al., 2019	ISS	140	MD: 1.6–4 cm	2	11	39	50	78	22–33	

*Measurements are for healthy subjects at rest and based on diffusion theory for homogeneous tissue, unless otherwise noted in the “Notes” column. Instr., Instrument; ISS, ISS, Inc. (Champaign, IL, United States); MD, multi distance; ECT, electro-convulsive therapy; TBI, Traumatic Brain Injury.*

Some studies have also reported the optical coefficients (*μ*_*a*_ and $\mu_{s}^{\prime }$) of brain tissue at discrete FD-NIRS wavelengths. This was done in newborn piglets (Fantini et al., 1999; Zhang et al., 2000), in neonates and infants (Zhao et al., 2005; Demel et al., 2014a; Farzam et al., 2017; Tian et al., 2017), and in adults (Hallacoglu et al., 2012; Blaney et al., 2019). Typically, optical properties were obtained by considering the tissue as homogeneous. However, a two-layer model has also been applied to analyze FD-NIRS data collected on adult human subjects to separately measure the optical properties of the superficial, extracerebral tissue (top layer) and the deeper brain tissue (bottom layer) (Hallacoglu et al., 2013). The absolute values of *μ*_*a*_ and $\mu_{s}^{\prime }$ reported in these studies are summarized in Table 4 and represent typical values (no error is provided) under baseline conditions.

**TABLE 4:** Absolute cerebral absorption and reduced scattering coefficients measured with non-invasive FD-NIRS *in vivo.*

References	Instr.	*f* (MHz)	Method	λ (nm)	*μ_*a*_* (cm^–1^)	*μ_*s*_’* (cm^–1^)	Age	Brain investigated
Fantini et al., 1999	ISS	110	MD: 1.5–3 cm	758	0.15	9.3	11 ± 1 days	Piglets
				830	0.13	8.2		
Zhang et al., 2000	ISS	110	MD: 1.5–3 cm	758	–	7.8	9 ± 2 days	Piglets
				830	–	6.6		
Zhao et al., 2005	Own	140	SD(calib): 4 cm	788	0.078	9.2	1–17 days	Neonates/infants/children
				832	0.089	8.4		
Demel et al., 2014a	ISS	110	MD: 1.5–3 cm	692	0.07–0.11	5.9–9.4	1–71 days	Neonates/infants/children
				834	0.08–0.12	4.5–7.2		
Farzam et al., 2017	ISS	110	MD: 1.5–3 cm	672	0.160	8.7	2 ± 1 days	Neonates/infants/children
				689	0.127	8.5		
				701	0.115	8.5		
				724	0.110	8.2		
				771	0.140	7.5		
				783	0.126	7.2		
				803	0.121	6.8		
				829	0.141	6.5		
Tian et al., 2017	ISS	110	MD	785, 811	0.15–0.30	6–18	0–16 years (ECMO)	Neonates/infants/children
Hallacoglu et al., 2012	ISS	110	MD: 2.8–3.8 cm	690	0.14	7.2	28 ± 4 years	Adults (homogeneous tissue)
				830	0.12	6.0		
				690	0.10	5.3	85 ± 6 years	
				830	0.08	4.8		
Hallacoglu et al., 2013	ISS	110	MD: 1.3–4.8 cm	690	0.08	13	29 ± 2 years	Adults (superficial tissue, ∼13 mm)
				830	0.08	11		
				690	0.20	3		Adults (deep tissue, brain)
				830	0.20	2		
				690	0.11	7		Adults (homogeneous tissue)
				830	0.12	5		
Blaney et al., 2019	ISS	140	MD: 1.1–4 cm	690	0.10	9.8	25–53 years	Adults (homogeneous tissue)
				830	0.11	8.0		

*Instr., Instrument; ISS, ISS, Inc. (Champaign, IL, United States); MD, multi distance; SD, single distance; ECMO, Extracorporeal Membrane Oxygenation.*

**FIGURE 2:**
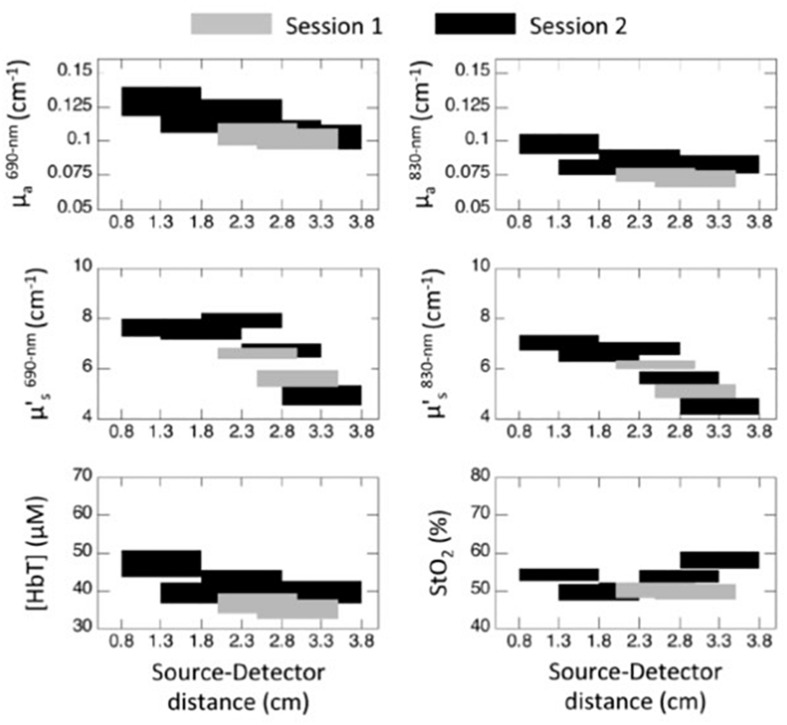
Absolute absorption coefficients (*μ*_*a*_: **top panels**) and reduced scattering coefficients ($\mu_{s}^{\prime }$: **middle panels**) measured with multi-distance FD-NIRS at 690 nm **(left panels)** and 830 nm **(right panels)** on the forehead of 16 elderly subjects (85 ± 6 years old) using diffusion theory for a homogeneous semi-infinite medium. From the absorption coefficients at two wavelengths, absolute values of concentration of hemoglobin ([HbT]) and tissue saturation (StO_2_) were obtained **(bottom panels)**. The blocks represent the mean ± standard error (vertical dimension) of the measurements performed at the range of distances corresponding to the horizontal range. The black and gray blocks correspond to two measurement sessions performed on the same group of 16 subjects 5 months apart. Reprinted with permission from Hallacoglu et al. (2012).

The optical properties measured with multi-distance FD-NIRS on the forehead of sixteen elderly subjects (85 ± 6 years old) by Hallacoglu et al., (2012) are reported in Figure 2 for different ranges of source-detector distance using diffusion theory for a homogeneous semi-infinite medium. Optical measurements were taken on the same group of subjects in two separate sessions separated in time by 5 months. The two measurement sessions are represented in Figure 2 by gray and black blocks, respectively. The source-detector distances employed in the first session were 2, 2.5, 3, and 3.5 cm, whereas in the second session they were 0.8, 1.3, 1.8, 2.3, 2.8, 3.3, and 3.8 cm. The horizontal extent of the blocks in Figure 2 indicates the distance range used for measurements of the optical properties according to the multi-distance method of Section “Multi-Distance Methods” (Fantini et al., 1999). The vertical extent of the blocks in Figure 2 represent the mean value ± the standard error of measurements on different subjects. Because data collected at longer distances are more sensitive to deeper tissue, the strong decrease in scattering vs. distance (i.e., the lower scattering values associated with the blocks representing measurements at longer source-detector distances in the middle panels of Figure 2) suggests that brain tissue may be less scattering than superficial scalp and skull tissue, even though the low scattering of the cerebrospinal fluid layer may also contribute to this result. This finding of a lower scattering of deeper tissue (subarachnoid space, brain, etc.) vs. superficial tissue (scalp, skull, etc.) is confirmed by a study that considered diffusion theory for a two-layered medium, and found a significantly lower scattering coefficient in the bottom tissue layer compared to the top tissue layer, whose thickness was found to be about 13 mm (Hallacoglu et al., 2013) (see Table 4).

In some cases, absolute measurements of baseline optical properties and hemoglobin concentrations obtained with multi-distance FD-NIRS are not reported, as the focus was the investigation of quantitative changes in [Hb] and [HbO_2_] associated with visual cortical activation in younger and older adults (McKernan Ward et al., 2015), brain activation with colored light (Scholkmann et al., 2017; Metz et al., 2017), a speech task (Scholkmann et al., 2013b, 2014a), or changes in cerebral blood flow (CBF) and StO_2_ following intrathoracic pressure strains (Zhang et al., 2017). Even in cases like these, where the emphasis is on measuring relative rather than absolute concentrations of hemoglobin, the ability of FD-NIRS to provide absolute measures of baseline optical properties can be beneficial. In fact, one does not need to rely on assumptions associated with the modified Beer–Lambert law in CW-NIRS, namely the values of the differential pathlength factor (DPF) at the wavelengths used (since the DPF can be obtained from the absolute optical properties), and the lack of changes in the scattering properties of tissue (since absolute scattering measurements allows one to verify whether scattering stays constant).

The absolute measurements of [HbT] (units: mol/l) afforded by FD-NIRS can be translated into absolute measurements of cerebral blood volume (CBV) [units: ml/(100 g)] by taking into account the concentration of hemoglobin in large blood vessels (HGB) (units: mol/l), the molecular weight of hemoglobin (MW_*Hb*_) (units: mol/g), the density of brain tissue (*D*_*bt*_) (units: g/ml), and the small-to-large vessel hematocrit ratio, or Fårhaeus factor (*F*) (Franceschini et al., 2007; Meng et al., 2012a):

(10) $$CBV=\frac{\left[HbT\right]\times MW_{Hb}}{HGB\times D_{bt}\times F}$$

Absolute measurements of StO_2_ (dimensionless) with multi-distance FD-NIRS have been combined with measurements of a cerebral blood flow index (BFI) with diffuse correlation spectroscopy (DCS) to yield a relative cerebral metabolic rate of oxygen (rCMRO_2_), with respect to a reference condition indicated by 0 subscripts (Roche-Labarbe et al., 2012):

(11) $$rCMRO_{2}=\frac{HGB\times BFI\times \left(SaO_{2}-StO_{2}\right)}{HGB_{0}\times BFI_{0}\times {\left(SaO_{2}-StO_{2}\right)}_{0}}$$

where SaO_2_ is the oxygen saturation of hemoglobin in arterial blood, and under the assumption of a constant relative contribution of arterial and venous blood to the StO_2_ measurement.

Using the approach of Eqs. (10) and (11), researchers have investigated the cerebral oxygen metabolism in neonates with intraventricular hemorrhage (Lin P. Y. et al., 2016) and the blood volume and metabolic responses to hand tactile stimulation in the somatosensory cortex of preterm neonates (Roche-Labarbe et al., 2014).

Single-distance methods (see Section “Single-Distance Methods”) can also yield absolute measurements, as long as they include a suitable calibration on a reference phantom with known optical properties. This method has been applied to the infant brain (Zhao et al., 2005).

### Stand-Alone Use of DC Data

There are research studies in the literature that are performed with FD-NIRS instrumentation, but report results obtained exclusively with DC intensity, which is equivalent to what would be measured in CW-NIRS. For example, this is the case for studies on the cerebral hemodynamics associated with auditory stimulation [concurrently with electro-encephalography (EEG)] (Tong et al., 2005), motor stimulation [concurrently with functional magnetic resonance imaging (fMRI)] (Sassaroli et al., 2006), different levels of mental workload (Sassaroli et al., 2008), cognitive multitasking (Solovey et al., 2011), different sleep stages (Pierro et al., 2012), a cycling exercise (Lin et al., 2013a), arterial blood pressure oscillations (Tgavalekos et al., 2016) and transients (Kainerstorfer et al., 2015) induced by pneumatic cuffs on the subject’s legs, electrical stimulation concurrent with voluntary movement (Lin T.-Y. et al., 2016), and fluctuations in intracranial pressure (Ruesch et al., 2019). One may wonder why the phase data was not considered in these studies. In some cases, it is possible that the most common objective of using phase data in FD-NIRS, namely their combination with intensity data to generate absolute measurements of optical properties, was not central to the study. In other cases, there may be reasons such as the lower signal-to-noise ratio of phase data, or uncertainties in how to exploit the added information content of phase data.

Some studies of brain functional activation have compared results obtained with stand-alone DC with those obtained with stand-alone phase (Sassaroli et al., 2004) or with combined DC and phase (Doulgerakis et al., 2019) to explore the importance of the additional information content of phase data. These studies concluded that phase data feature greater sensitivity to deeper, i.e., cerebral, tissue compared to DC data. This point is further explored in Section “Enhanced Depth Sensitivity Using Phase Data” on the depth sensitivity of stand-alone phase measurements.

### Stand-Alone Use of AC Data

**FIGURE 3:**
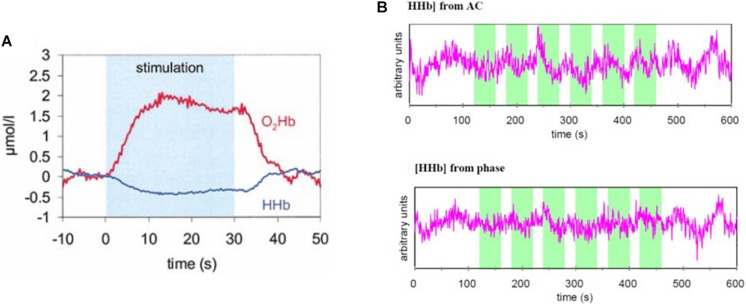
Functional FD-NIRS on the human brain. **(A)** Hemodynamic response to visual stimulation (checkerboard reversing) measured in the human visual cortex with AC data at two wavelengths (758 and 830 nm) translated into concentration changes in oxyhemoglobin (O_2_Hb) and deoxyhemoglobin (HHb). **(B)** Deoxyhemoglobin concentration ([HHb]) traces obtained from AC data (top) and phase data (bottom) in the human prefrontal cortex in a protocol involving arithmetic calculations (indicated by the green shaded areas in the two panels. **(A)** Reprinted with permission from Wolf et al. (2002b) Elsevier; **(B)** Reprinted with permission from Toronov et al. (2001) The Optical Society of America.

While the information content of AC data is essentially the same as for DC data, at least at modulation frequencies typically used in FD-NIRS (say, <150 MHz), the AC amplitude is weakly affected by room light or stray light, which instead may significantly impact the DC intensity. For this reason, some researchers opted to use frequency-domain instrumentation to collect AC data that is then analyzed using data processing techniques typically associated with CW-NIRS (modified Beer–Lambert law with assumed values of DPF). This approach has been used for brain-computer interface research in the areas of decision making (Luu and Chau, 2009), classification of prefrontal activity (Power et al., 2010), for the assessment of independent component analysis (ICA) and principal component analysis (PCA) in removing extracerebral tissue contributions to the NIRS signal (Virtanen et al., 2009), and to investigate the hemodynamics in the prefrontal cortex during arithmetic calculations (Toronov et al., 2001), in the frontal cortex during breath holding, hyperventilation, and sleep (Noponen et al., 2003), in the motor and visual cortices during functional activation (Wolf et al., 2002a,b), and in the visual cortex under normo-, hypo-, and hypercapnia conditions (Noponen et al., 2005). Figure 3A shows the hemodynamic response to visual stimulation by checkerboard reversing in the human visual cortex measured with AC data, showing the typical increase in oxyhemoglobin and decrease in deoxyhemoglobin associated with the activation-induced increase in cerebral blood flow (Wolf et al., 2002b). The top panel of Figure 3B shows the decrease in deoxyhemoglobin concentration, measured with AC data, in the human prefrontal cortex as a result of arithmetic calculations (Toronov et al., 2001). Note the latency of several seconds for the hemodynamic response to brain activation.

While CW-NIRS data are conceptually equivalent to DC data in FD-NIRS, it is worth noting that it is common for CW-NIRS instruments to use frequency-encoding approaches to distinguish data from different light sources. While the modulation frequency in these cases is on a scale of kHz, thus inappropriate for FD-NIRS, it nevertheless results in a suppression of room light contributions, similarly to AC data in FD-NIRS.

### Stand-Alone Use of Phase Data

While the signal-to-noise ratio associated with phase data in FD-NIRS is typically lower than that of DC and AC data, the unique information content of phase data and the different region of sensitivity of phase vs. DC or AC data (see Figure 1) justify a careful consideration of stand-alone phase. Regarding signal-to-noise ratio, it has been argued that higher modulation frequencies in the range 400–500 MHz may result in a significant improvement over phase measurements at the typical modulation frequencies of ∼100 MHz used in FD-NIRS (Toronov et al., 2004).

Stand-alone phase data have been used for quantitative brain oximetry (i.e., measurements of StO_2_), after assuming values for the reduced scattering coefficient of tissue (Sevick et al., 1991; Wilson et al., 1992), or after calibration on known phantoms (Kurth and Thayer, 1999). These methods were applied to newborn piglets during a hypoxic challenge (Ntziachristos et al., 1997), and to human newborns and children with congenital heart disease (Watzman et al., 2000) (see Tables 1, 2).

In the case of fNIRS, one is typically interested in hemodynamic changes associated with brain activation. Such hemodynamic changes affect both intensity and phase data and are mostly associated with absorption changes due to blood volume and blood oxygenation dynamics induced by brain activity. Under the assumption of negligible scattering changes, temporal phase data can be translated into measurements of Δ[Hb] and Δ[HbO_2_] (concentration changes with respect to baseline) (Toronov et al., 2001; Blaney et al., 2020) [see Eq. (6)], similar to the way intensity changes are translated into Δ[Hb] and Δ[HbO_2_] by the modified Beer-Lambert law in CW NIRS (Delpy et al., 1988; Sassaroli and Fantini, 2004) [see Eq. (5)]. Phase-only measurements on the human prefrontal cortex during a protocol involving arithmetic calculations (Toronov et al., 2001) and on the primary motor cortex during a finger-tapping task (Sassaroli et al., 2004) demonstrated the ability of stand-alone phase data to detect brain activation. The bottom panel of Figure 3B shows the decrease in deoxyhemoglobin concentration, measured with phase data, in the human prefrontal cortex as a result of arithmetic calculations (Toronov et al., 2001).

### The Fast Optical Signal

**FIGURE 4:**
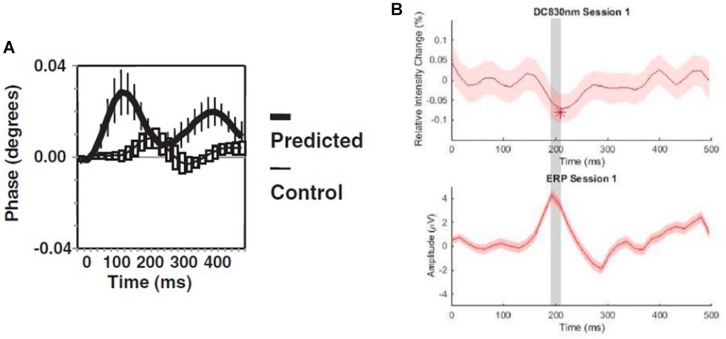
Fast optical signal measured by FD-NIRS with **(A)** phase from the visual cortex of human subjects at the cortical location of predicted response and at a control location (EROS: event-related optical signal), and **(B)** DC Intensity from the prefrontal cortex (top) in comparison with the event-related potential (ERP) response. **(A)** Reprinted with permission from Gratton and Fabiani (2003)© Wiley; **(B)** Reprinted with permission from Proulx et al. (2018b)© Elsevier.

FD-NIRS has been applied to the study of the so-called fast optical signal (occurring on a time scale of ∼100 ms), which is thought to be associated with scattering changes that are more directly representative of neuronal activation than the absorption changes associated with cerebral hemodynamic responses (occurring on a time scale of seconds). The fast optical signal was first reported with phase measurements in the human visual cortex under the name EROS, for “event-related optical signal” (Gratton et al., 1995a). Phase measurements, and sometimes AC measurements, were then used to investigate the fast optical signal in the visual (Gratton and Fabiani, 2003; Gratton et al., 2006; Maclin et al., 2007), auditory (Maclin et al., 2003), somatosensory (Maclin et al., 2004), motor (Gratton et al., 1995b; Parks et al., 2012), temporal/frontal (Tse et al., 2006, 2013; Tse and Penney, 2007), and prefrontal (Low et al., 2006, 2009; Gratton et al., 2009; Huang et al., 2013; Baniqued et al., 2013) cortices. Brain activation was reported to evoke a fast, transient phase increase of the order of 0.05° (sometimes extending to values of 0.1° or 0.2°) (see Figure 4A).

Stand-alone DC data, AC data, and phase data have been considered in the investigation of the fast optical signal. Some human studies on the motor cortex (Wolf et al., 2002a; Morren et al., 2004), visual cortex (Wolf et al., 2003), and prefrontal cortex (Proulx et al., 2018a, b) reported that DC and AC data can detect the fast signal more effectively than phase data. The fast optical signal was also investigated with CW-NIRS in the somatosensory cortex (Steinbrink et al., 2000), prefrontal cortex (Medvedev et al., 2010), temporal cortex (Kubota et al., 2008), visual cortex (Sun et al., 2014), and motor cortex (Zhang et al., 2012). However, given the small amplitude of the fast optical signal, questions were raised about its detectability (Steinbrink et al., 2005). In fact, the fast optical signal consists of a small transient decrease in the DC intensity (or AC amplitude), of the order of 0.05% or less (see Figure 4B). (We note that an animal study on the somatosensory cortex of marmosets found a transient *increase* of ∼0.08% in the CW intensity, in a method termed optoencephalography; Hiwaki and Miyaguchi, 2018). The challenge of detecting the fast optical signal was further demonstrated in studies of the human motor cortex (Franceschini and Boas, 2004) and the primate visual cortex (Radhakrishnan et al., 2009).

### Enhanced Depth Sensitivity Using Phase Data

**FIGURE 5:**
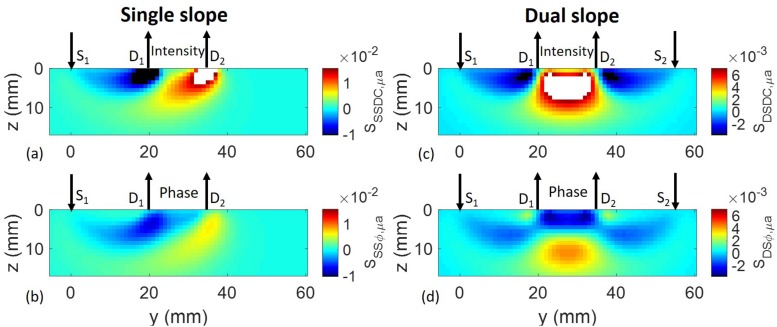
Sensitivity maps for absorption contrast of **(A)** single-slope intensity, **(B)** single-slope phase, **(C)** dual-slope intensity, and **(D)** dual-slope phase. The arrows pointing down indicate the light sources (S_1_, S_2_), and the arrows pointing up indicate the optical detectors (D_1_, D_2_). The color bar labels in panels **(A,C)** indicate the sensitivity to absorption perturbations for single slope DC intensity (*S*_*SSDC*,*μ*_*a*__) and dual slope DC intensity (*S*_*DSDC*,*μ*_*a*__), respectively. The color bar labels in panels **(B,D)** indicate the sensitivity to absorption perturbations for single slope phase (*S*_*SSφ*,*μ*_*a*__) and dual slope phase (*S*_*DSφ*,*μ*_*a*__), respectively. White and black in the color maps indicate values greater than the maximum or smaller than the minimum, respectively, of the color bars. Adapted with permission from Sassaroli et al. (2019)© The Optical Society of America.

A comparison of Figures 1A,B shows that single-distance phase measurements feature a sensitivity to absorption perturbations that extends deeper than single-distance intensity measurements. In fact, as already mentioned, it was observed that phase measurements provide deeper sensitivity, and thus a stronger sensitivity to cerebral tissue in non-invasive optical studies of the human brain (Sassaroli et al., 2004; Doulgerakis et al., 2019). In an effort to minimize the sensitivity to superficial tissue layers, multi-distance measurements were introduced in both CW and FD-NIRS, and implemented with either a single source and multiple detectors, or a single detector and multiple sources (Franceschini et al., 1998; Suzuki et al., 1999). Since these methods ultimately aimed at measuring a gradient, or slope, of optical signals versus source-detector distance, we refer to these methods as *single-slope* methods. These methods have been extensively used in both CW-NIRS and FD-NIRS, and have been implemented into several commercial NIRS instruments. While single-slope methods indeed feature a small sensitivity to uniform absorption changes in superficial layers, they may yield misleading results in the case of localized superficial perturbations. In fact, as shown in Figures 5A,B, the single-slope sensitivity of both intensity and phase feature double, asymmetrical banana-shaped regions with sensitivities of opposite sign (Sassaroli et al., 2019). This feature may be problematic in the case of localized superficial inhomogeneous changes in optical properties.

We have recently proposed to enhance depth sensitivity using a particular source-detector arrangement that was originally introduced for self-calibrating, absolute measurements of optical properties with FD-NIRS (Hueber et al., 1999) (as described in Section “Multi-Distance Methods”). This particular arrangement involves at least two sources *and* at least two detectors to measure two paired gradients or slopes of optical signals versus source-detector distance, which are then averaged. Because it is based on the measurement of two slopes, this approach is referred to as a *dual-slope* method, and it can be applied separately to intensity and phase data. The region of sensitivity of dual slopes for intensity and phase, in the case of a linear arrangement of two sources and two detectors, is illustrated in Figures 5C,D for the same optical properties and modulation frequency of Figure 1. In this case, however, we considered a larger absorption perturbation having an axial size (along *z*) of 1 mm and a lateral size (across *x* and *y*) of 5 mm, which may represent a typical size of localized hemodynamic changes (as related to functional brain activation or extracerebral hemodynamics). A variety of other source-detector arrangements for dual-slope measurements, and the corresponding intensity and phase regions of sensitivity, have also been reported (Fantini et al., 2020). The dual-slope sensitivity features both positive and negative values, and takes maximum values deeper in the tissue than the single-distance sensitivity (in the case of Figure 5: ∼5 mm vs. ∼2 mm for intensity, and ∼11 mm vs. ∼5 mm for phase). Furthermore, it is evident from Figures 5C,D that the sensitivity of phase dual-slope extends deeper than the sensitivity of intensity dual-slope. An FD-NIRS study on human subjects showed the different information content of intensity and phase collected with single-distance, single-slope, and dual-slope arrangements, consistent with a deeper sensitivity of phase slope methods, and more robust dual-slope vs. single-slope data (Blaney et al., 2020).

The above observations about single-slope and dual-slope sensitivities have also been articulated in the time domain, where source-detector arrangements for self-calibrating absolute measurements have been shown to achieve a better depth sensitivity with the first and second moments of the photon time-of-flight distribution (the first moment is representative of the phase in the frequency domain), with respect to the 0th moment (which represents the CW intensity) (Sawosz and Liebert, 2019).

## Critical Perspective: What Is the Major Promise of FD Data in Cerebral NIRS?

FD-NIRS provides data with richer information content than CW-NIRS. In this article, we have discussed how such additional information may be used in three broad areas: (1) absolute quantification of baseline optical properties and hemoglobin concentration in tissue; (2) minimization of sensitivity to room light and other potential sources of confounds (such as instrumental drifts, variable optical coupling with tissue, and motion artifacts) in practical measurements; (3) achievement of selective sensitivity to deeper tissue.

The ability to perform absolute baseline measurements is undoubtedly appealing, since it leads to the possibility of determining normative baseline values, performing comparisons across subjects, and conducting longitudinal studies over long periods of time. However, the accuracy of absolute measurements may be significantly affected by simplifying assumptions (on the tissue geometry, spatial distribution of optical properties, etc.), and by instrumental or experimental artifacts that are often difficult to avoid in real-life measurement conditions. These assumptions and experimental artifacts can be particularly limiting in the case of non-invasive brain studies because of the strong tissue heterogeneity, presence of hair, tissue rigidity due to the skull bone, etc. Furthermore, many applications of functional NIRS or cerebral oximetry do not need an absolute baseline characterization, but rather rely on relative changes during specific measurement protocols.

Practical advantages related to a lack of sensitivity to room light or other sources of instrumental artifacts are important, but may also be achieved with alternative methods.

In our opinion, the one aspect of FD-NIRS that holds the most promise to advance the field of non-invasive optical sensing of the brain is the ability to control the region of sensitivity by taking advantage of intensity and phase measurements collected with properly designed source-detector arrays. Such a capability is not available to CW-NIRS, and can help address one of the most critical open questions in the field, namely the minimization of confounding contributions from extracerebral tissue (scalp, skull, etc.) to non-invasive optical measurements of the brain. The possibility of achieving more selective sensitivity to brain tissue, while also providing access to valuable absolute measurements of optical properties and chromophore concentrations, renders FD-NIRS a powerful technique for non-invasive measurements of the human brain.

## Author Contributions

SF and AS: literature survey, analysis, and writing. SF: tables.

## Conflict of Interest

The authors declare that the research was conducted in the absence of any commercial or financial relationships that could be construed as a potential conflict of interest.
